# Mechanism and Parameter Optimization of Fenton’s Reagent Integrated with Surfactant Pretreatment to Improve Sludge Dewaterability

**DOI:** 10.1371/journal.pone.0169523

**Published:** 2017-01-12

**Authors:** Yi Xing, Zhiqiang Wang, Chen Hong, Qiang Yang, Lihui Feng, Mengmeng Jia, Yifei Li

**Affiliations:** 1 Department of Environmental Engineering, University of Science and Technology Beijing, Beijing, China; 2 Beijing Key Laboratory of Resource-oriented Treatment of Industrial Pollutants, University of Science and Technology Beijing, Beijing, China; 3 Reasearch Center for Eco-Environmental Sciences, Chinese Academy of Science, Beijing, China; Peking University, CHINA

## Abstract

Sludge dewatering can effectively reduce the volume and mass of sludge for subsequent treatment and disposal. The work validated the potential of Fenton’s reagent combined with dodecyl dimethyl benzyl ammonium chloride (DDBAC) in improving sludge dewaterability and proposed the mechanism of joint conditioning. The composite conditioner dosage was optimized using response surface methodology. Results indicated the good conditioning capability of the composite conditioners. The optimum dosages for H_2_O_2_, Fe^2+^, and DDBAC were 44.6, 39.6, and 71.0 mg/g, respectively, at which a sludge cake water content of 59.67% could be achieved. Moreover, a second-order polynomial equation was developed to describe the behavior of joint conditioning. Analysis of the reaction mechanism showed that Fenton oxidation effectively decomposed extracellular polymeric substance (EPS), including loosely bound EPS (LB-EPS) and tightly bound EPS (TB-EPS), into dissolved organics, such as proteins and polysaccharides. The process facilitated the conversion of the bound water into free water. Furthermore, DDBAC further released the bound water through solubilization of TB-EPS and LB-EPS after the Fenton reaction. The bound water content of the sludge conditioned with Fenton’s reagent decreased from 3.15 to 1.36 g/g and further decreased to 1.08 g/g with the addition of DDBAC. High-performance liquid chromatography analysis verified that the composite conditioning could oxidize and hydrolyze EPS into low-molecular-mass organics (e.g., formic and acetic acid), thereby facilitating the release of bound water.

## Introduction

Sewage sludge, a by-product of wastewater treatment, contains a large amount of water that can be generally divided into free and bound water [1–3]. Free water is unrestrained by sludge solid particles and can be easily separated from the sludge by using mechanical stress, whereas the bound water is difficult to be removed using the mechanical dewatering methods because of the presence of chemical bonds between bound water and sludge flocs [4]. Thus, sludge conditioning and dewatering are essential to improve sludge dewaterability. Organic polymers can facilitate the flocculation of sludge particles by charge neutralization or adsorption bridging mechanism but do not affect the water molecule-organic matter bonding in the sludge. Moreover, the complicated degradability of residual polymer in sludge cakes cause secondary environmental pollution.

The Fenton system generates hydroxyl radicals (·OH) with powerful oxidizing abilities through iron-catalyzed H_2_O_2_ decomposition under acidic conditions. ·OH can decompose organic matter and rupture microbial cells [5,6]. Researchers [7–10] have concluded that Fenton’s reagent can improve sludge dewaterability. Liu et al. [7] demonstrated that 84% specific resistance to filtration (SRF) reduction efficiency was achieved when 20 mg/g Fe^2+^ and 125 mg/g H_2_O_2_ were added to the sludge. Moreover, their studies [8–10] reported that the addition of Fenton’s reagent increases the extracellular polymeric substance (EPS) concentration in the sludge supernatant, leading to significant effects on sludge dewatering performance.

Extracellular polymeric substance, which mainly consists of proteins and polysaccharides, is an insoluble organic matter attached to the bacterial cell surface and is closely related to the bound water [11–13]. According to the combined extent between organic matter and sludge flocs, EPS can be divided into slime layer EPS (S–EPS), loosely bound EPS (LB–EPS), and tightly bound EPS (TB–EPS) [14]. Researchers have reported that cationic surfactants (CTAB and DTAB) could be used to improve sludge filtration efficiency and decrease sludge cake water content substantially [15–17]. However, the effects of the cationic surfactant dodecyl dimethyl benzyl ammonium chloride (DDBAC) on sludge dewatering performance have been rarely investigated. Our previous studies [18,19] revealed that DDBAC can effectively release bound water from sludge because of its superior surface activity and strong adsorption/bridge capacities on sludge. Moreover, the application of Fenton’s reagent integrated with DDBAC during sludge conditioning has not been investigated. Furthermore, all the above-mentioned studies focused on the relationship between S–EPS concentration and sludge dewatering performance. The effects of composite conditioners on LB–EPS and TB–EPS, and the relationship between EPS (S–EPS, LB–EPS, and TB–EPS) and bound water during joint conditioning are still unclear. In addition, optimal composite conditioner dosages have not been concluded yet.

In this study, water content of sludge cake (*W*_C_) and capillary suction time (CST) were used as sludge dewaterability indicators. Composite conditioner dosages were optimized using single-factor experiments and response surface methodology (RSM). In addition, bound water content (*W*_B_), EPS concentration, and organic acid concentrations in the sludge supernatant were determined to clarify the mechanism of Fenton’s reagent integrated with surfactant pretreatment to improve sludge dewaterability.

## Materials and Methods

### Test materials

The sludge used in this study was collected from secondary sedimentation tank of Xiaohongmen municipal wastewater treatment plant in Beijing, China. The samples were thickened by gravity to increase the concentration and stored in polypropylene containers at 4°C before use. All experiments were completed in 72 h. The main characteristics of the thickened sludge are listed in Table 1.

**Table 1:** Characteristics of the thickened sludge before conditioning.

Parameters		Value
**TSS**^a^ **(mg/L)**		46583.91 ± 362.57
**VSS**^b^ **(mg/L)**		31199.43 ± 265.68
**pH**		7.23 ± 0.11
**Water content (%)**		95.11 ± 0.24
**Bound water content (g/g)**		3.18 ± 0.09
**S-EPS**	**Protein (mg/L)**	363.31 ± 16.82
	**Polysaccharide (mg/L)**	72.68 ± 5.08
**LB-EPS**	**Protein (mg/L)**	143.16 ± 7.57
	**Polysaccharide (mg/L)**	29.06 ± 2.13
**TB-EPS**	**Protein (mg/L)**	3028.17 ± 169.65
	**Polysaccharide (mg/L)**	593.59 ± 26.27

^a^ total suspended solids;

^b^ volatile suspended solids

Sulfuric acid (H_2_SO_4_, 4 M) was used to adjust the initial pH of sludge to 4 before adding Fenton’s reagent. Fenton’s reagent was prepared by mixing FeSO_4_·7H_2_O and H_2_O_2_ (30 wt%). The cationic surfactant was DDBAC with a chemical formula of C_21_H_38_NCl and relative molecular mass of 340.00. All the chemicals used were of analytical grade, except for the bovine serum albumin of biochemical grade, and were purchased from Sinopharm Chemical Reagent Company, China.

### Experimental design

#### Single factor experiments

The effects of H_2_O_2_ (from 10 to 100 mg/g) on the *W*_C_ and CST were investigated with 20 and 40 mg/g of Fe^2+^ and DDBAC, respectively. Similarly, the effects of Fe^2+^ (from 10 to 100 mg/g) on the *W*_C_ and CST were studied with 40 and 40 mg/g of H_2_O_2_ and DDBAC, respectively. The effects of DDBAC (from 0 to 100 mg/g) on the *W*_C_ and CST were evaluated with 40 and 40 mg/g of H_2_O_2_ and Fe^2+^.

#### RSM design

A Box-Behnken design [20] was chosen to evaluate the combined effects of H_2_O_2_, Fe^2+^, and DDBAC during conditioning. The range and levels of the three variables were defined according to the single-factor experiments and are presented in Table 2. Moreover, *W*_C_ was examined as the response value. Seventeen runs were required for a complete set of the experimental design, as shown in Table 3, and the experimental results were analyzed using Design-Expert 8.0. The response variable (*Y*) was connected with the set of the independent variables using an empirical second-order polynomial model. The generalized model is in the following form [20]:

$$Y=\beta_{0}+\overset{3}{\underset{i=1}{\sum }}\beta_{i}X_{i}+\overset{3}{\underset{i=1}{\sum }}\beta_{i}{}_{i}X_{i}^{2}+\sum^{\;}\overset{3}{\underset{i<\text{j}=2}{\sum }}\beta_{i}{}_{\text{j}}X_{i}X_{\text{j}}$$ (1)

where *Y* is the predicted response (*W*_C_, %); *β*_0_, *β*_*i*_, *β*_*ii*_, and *β*_*i*j_ are defined as the model regression coefficients; and *X*_i_ and *X*_j_ are the independent variables.

**Table 2:** Range and levels of the variables in Box-Behnken design.

Variable (mg/g)	Range and levels		
	-1	0	1
***X***_**1**_, **H**_**2**_**O**_**2**_ **dosage**	20	40	60
***X***_**2**_, **Fe**^**2+**^ **dosage**	20	40	60
***X***_**3**_, **DDBAC dosage**	40	60	80

**Table 3:** RSM for the three experimental variables in coded units.

Run No.	Coded Factors			Run No.	Coded Factors			Run No.	Coded Factors		
	*X*_*1*_	*X*_*2*_	*X*_*3*_		*X*_*1*_	*X*_*2*_	*X*_*3*_		*X*_*1*_	*X*_*2*_	*X*_*3*_
**1**	-1	-1	0	**7**	-1	0	1	**13**	0	0	0
**2**	1	-1	0	**8**	1	0	1	**14**	0	0	0
**3**	-1	1	0	**9**	0	-1	-1	**15**	0	0	0
**4**	1	1	0	**10**	0	1	-1	**16**	0	0	0
**5**	-1	0	-1	**11**	0	-1	1	**17**	0	0	0
**6**	1	0	-1	**12**	0	1	1				

### Sludge conditioning and dewatering

Approximately 300 mL of the thickened sludge samples were poured into a 500 mL beaker and conditioned according to the following procedures: addition of 4 M H_2_SO_4_ → rapid mixing at 200 rpm for 1 min → addition of Fe^2+^ solution → rapid mixing at 200 rpm for 1 min → addition of H_2_O_2_ → slow mixing at 100 rpm for 120 min → addition of 4 M NaOH to neutralize the pH of sludge samples to approximately 7 (terminating the Fenton reaction) → addition of DDBAC → slow mixing at 100 rpm for 30 min.

After conditioning, 50 mL conditioned sludge sample was filtered and dewatered in a 150 mm standard Buchner funnel with a quantitative filter paper at a constant vacuum pressure of −0.055 MPa until no filtrate came out.

### Sludge dewaterability

Sludge dewaterability was evaluated by determining *W*_C_ and CST. *W*_C_ was calculated using the equation:

$$W_{\text{C}}=\left(W_{1}-W_{2}\right)/W_{1}\times 100\%$$ (2)

where *W*_1_ is the sludge cake weight (g), and *W*_2_ is the sludge cake weight after drying to a constant weight at 105°C (g).

CST was measured using a standard CST apparatus (304 M, Triton). The pH value of the sludge was determined using a digital pH-meter (FE20, METTLER-TOLEDO).

### Conditioning mechanism investigation

As shown in Table 4, a set of experiments with different conditioning preparations were conducted to elucidate the mechanism of the composite conditioner pretreatment to improve sludge dewaterability. The sludge before and after conditioning was analyzed in terms of EPS concentration, *W*_B_ and organic acid concentrations in the sludge supernatant.

**Table 4:** Different conditioning preparations for sludge.

Sludge	Symbol	pH	Dosage (mg/g)		
			H_2_O_2_	Fe^2+^	DDBAC
**Raw sludge**	RS	7.23	0	0	0
**Sludge conditioned by Fenton’s reagent**	F	4	44.6	39.6	0
**Sludge conditioned by DDBAC**	D	4	0	0	71.0
**Sludge conditioned by Fenton’s reagent and DDBAC**	FD	4	44.6	39.6	71.0

#### EPS extraction and analysis

EPS samples were extracted according to our previous studies [15]. All the S-EPS, LB-EPS, and TB-EPS extractions were analyzed for proteins (PN) and polysaccharides (PS). The PN concentration was determined based on the method of Lowry et al. [21] using bovine serum albumin as the standard; whereas the PS concentration was measured according to the method of Riesz et al. [22] using glucose as the standard.

#### Bound water content measurement

The *W*_B_ was measured using a differential scanning calorimetry (DSC) analyzer (404 F3 Pegasus, NETZSCH) equipped with a liquid nitrogen cooling system. The sludge cake sample was first cooled to -20°C, assuming that all free water was frozen at this temperature, and then warmed to 20°C at the rate of 2°C/min [23]. The DSC curve showed an endothermic peak during the free water transition. Thus, the free water can be calculated using the area of the endothermic curve below the baseline. *W*_B_ was determined by the difference between total and free water in the sludge cake:

$$W_{\text{B}}=W_{\text{T}}-△H/△H_{0}$$ (3)

where *W*_B_ is the bound water content (g/g, is the matter content in per gram of dry sludge, based on dry solids, similarly hereinafter), *W*_T_ is the total water content (g/g), *ΔH* is the endothermic curve area of the sample per gram of dry solid (J/g), and *ΔH*_0_ is the standard fusion heat of ice at 334.7 J/g.

#### HPLC study

Organic matter hydrolysis in the sludge supernatant was analyzed using HPLC (LC-20AD, Shimadzu Corp., Japan). HPLC was applied to determine the concentration of small molecular organic acids (i.e., formic, acetic, and propionic acids).

HPLC was performed under the following conditions: the mobile phase was 7% CH_3_OH-0.20 mol/L KH_2_PO_4_ (pH = 4.0) buffer solution (v/v) filtered with a 0.45 μm membrane and then degassed by ultrasonication for 10 min. The flow rate was 0.5 mL/min, the column temperature was 40 ± 1°C, the measurement wavelength was 215 nm, the injection volume was 20 μL, and the high peaks were quantified using the external standard method.

## Results and Discussion

### Single factor experiments to determine the optimum range of three factors

**Fig 1:**
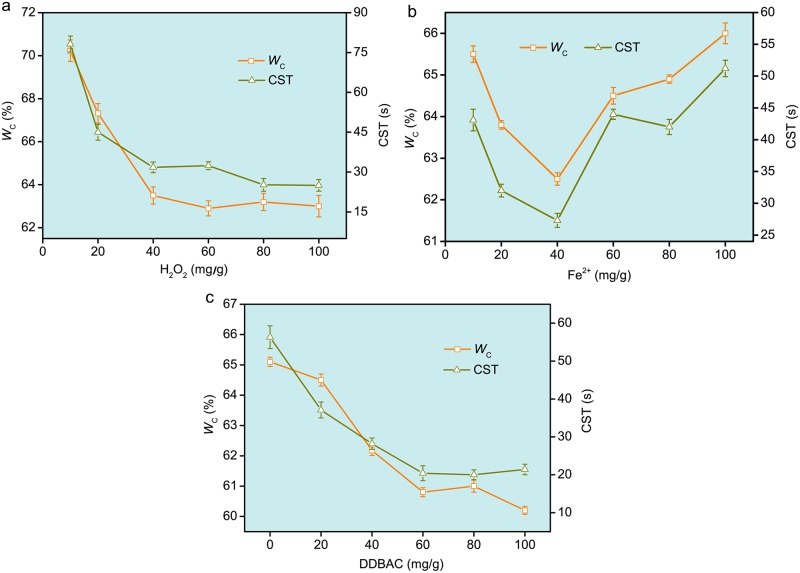
Effects of the composite conditioner of Fenton’s reagent and DDBAC on the sludge dewaterability. a Fe^2+^ dosage 20 mg/g; H_2_O_2_ dosage 10–100 mg/g; DDBAC dosage 40 mg/g. b Fe^2+^ dosage 10–100 mg/g; H_2_O_2_ dosage 40 mg/g; DDBAC dosage 40 mg/g. c Fe^2+^ dosage 40 mg/g; H_2_O_2_ dosage 40 mg/g; DDBAC dosage 0–100 mg/g.

Fig 1 shows that the effects of the composite conditioner on sludge dewaterability. The *W*_C_ and CST decreased to 63.36% and 31.8 s (Fig 1a), respectively, when the H_2_O_2_ dosage was increased to 40 mg/g (mg/g, being the H_2_O_2_ dosage per gram of dry sludge, similarly hereinafter). This was likely due to the fact that ·OH concentration increased as H_2_O_2_ dosage increased, thereby enhancing the capacity of ·OH in oxidizing the sludge [24]. However, H_2_O_2_ could react with ·OH and form hydroperoxyl radicals (HO_2_·) whose oxidizing capacity is inferior to that of ·OH [24,25]. Thus, the decreasing trends of *W*_C_ and CST slowed down as H_2_O_2_ dosage further increased. The optimum H_2_O_2_ dosage was thought to be approximately 40 mg/g. The finding was in agreement with that reported by Zhou et al. [5], who observed that adding more H_2_O_2_ cannot further improve the sludge dewaterability.

Similarly, the effects of Fe^2+^ on the sludge dewaterability are shown in Fig 1b. The *W*_C_ and CST of the sludge decreased as Fe^2+^dosage increased (>10 mg/g) and the lowest *W*_C_ (62.50%) and CST (27.3 s) were obtained at 40 mg/g of Fe^2+^. This is mainly because Fe^2+^ was a catalyst in the Fenton reaction system and could generate ·OH after reacting with H_2_O_2_. Thus, ·OH could be increased with the increase of Fe^2+^ dosage. The ·OH oxidized sludge flocs and microorganisms, which boosted the sludge dewatering performance by releasing the bound water into the liquid phase [26]. However, with a further increase in Fe^2+^ dosage, the *W*_C_ and CST rapidly increased. The excess Fe^2+^ (>40 mg/g) could consume the ·OH, thereby producing the non-oxydic OH^−^ [6]. The concentration of oxydic ·OH decreased compared with that at 40 mg/g of Fe^2+^ dosage, thereby decreasing the treatment efficiency.

The effects of DDBAC on sludge dewatering performance, as depicted in Fig 1c, were obvious and similar in effect to that of H_2_O_2_ (Fig 1a). The *W*_C_ and CST decreased as DDBAC dosage increased (≤60 mg/g). After that, sludge dewatering performance was almost unchanged. The results coincided with those in our previous studies [18,19]. Thus, the optimum DDBAC dosage is approximately 60 mg/g.

### Optimization of the composite conditioner using RSM

**Fig 2:**
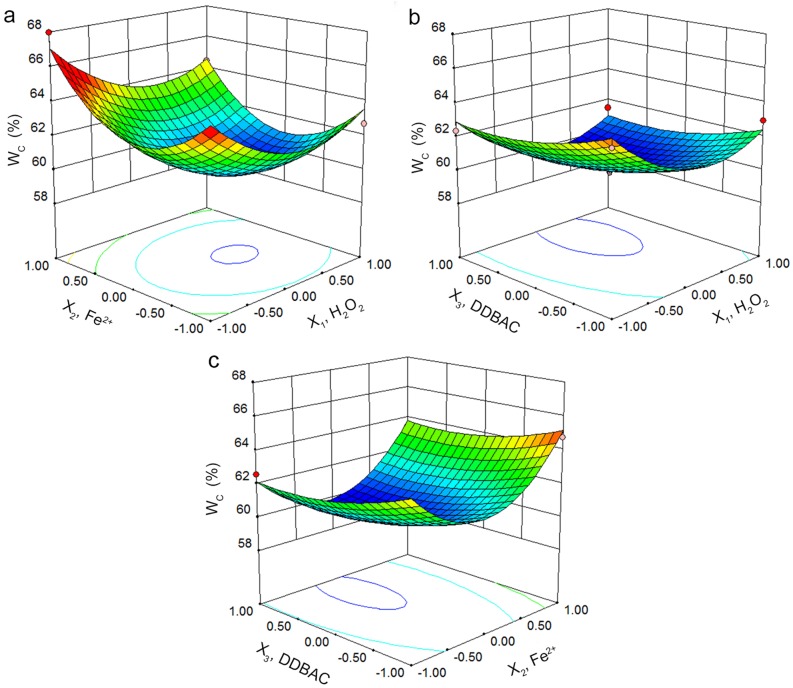
3D surface graphs and contour plots of *W*_C_ showing the effects of variables. a *X*_1_-*X*_2_. b *X*_1_-*X*_3_. c *X*_2_-*X*_3_.

The three-dimensional surface and contour plots of the response *W*_C_, obtained using Design-Expert 8.0, visualized the predicted model. Fig 2a–2c display the relationship between two interacting variables with the response when the third variable was kept at its zero level. In Fig 2a, an obvious decrease of *W*_C_ was observed as H_2_O_2_ dosage increased. *W*_C_ was almost unchanged when the H_2_O_2_ dosage was at higher value, which agreed with the single factor experimental results (Fig 1a). Similarly, the *W*_C_ decreased as Fe^2+^ dosage increased within a certain limit. After that, the increasing Fe^2+^ severely deteriorated sludge dewatering performance, as described above (Fig 1b). The 3D surface and the corresponding contour plotted in Fig 2b illustrate that combining H_2_O_2_ and DDBAC significantly affected the *W*_C_. Similarly, Fig 2c shows the effects of Fe^2+^ and DDBAC on the *W*_C_ in 40 mg/g H_2_O_2_. Hence, optimizing the H_2_O_2_, Fe^2+^, and DDBAC dosages was performed to achieve the lowest *W*_C_ from the statistical point of view.

Table 5 presents the results of the experiments based on the Box-Behnken design. After data fitting, a second-order polynomial equation was obtained as:

$$\begin{array}{l} W_{\text{C}}=60.02-0.96X_{1}+0.77X_{2}-0.71X_{3}-0.26X_{1}X_{2}+0.02X_{1}X_{3}+0.05X_{2}X_{3}\\ +1.99X_{1}^{2}+3.04X_{2}^{2}+0.65X_{3}^{2} \end{array}$$ (4)

**Table 5:** The experimental and predicted responses of the composite conditioner in conditioning wastewater sludge.

Run No.	Coded Factors			Response(*W*_C_)	
	*X*_*1*_	*X*_*2*_	*X*_*3*_	Experimental	Predicted
**1**	-1	-1	0	65.11 ± 0.32	64.98 ± 0.34
**2**	1	-1	0	62.67 ± 0.34	63.57 ± 0.62
**3**	-1	1	0	67.94 ± 0.40	67.04 ± 0.12
**4**	1	1	0	64.48 ± 0.24	64.60 ± 0.32
**5**	-1	0	-1	63.87 ± 0.37	64.36 ± 0.22
**6**	1	0	-1	62.93 ± 0.32	62.40 ± 0.47
**7**	-1	0	1	62.36 ± 0.17	62.89 ± 0.14
**8**	1	0	1	61.50 ± 0.27	61.01 ± 0.46
**9**	0	-1	-1	64.07 ± 0.21	63.70 ± 0.25
**10**	0	1	-1	64.75 ± 0.25	65.16 ± 0.40
**11**	0	-1	1	62.59 ± 0.33	62.18 ± 0.13
**12**	0	1	1	63.45 ± 0.28	63.82 ± 0.45
**13**	0	0	0	60.25 ± 0.22	60.02 ± 0.32
**14**	0	0	0	60.03 ± 0.39	60.02 ± 0.32
**15**	0	0	0	59.84 ± 0.27	60.02 ± 0.32
**16**	0	0	0	59.92 ± 0.37	60.02 ± 0.32
**17**	0	0	0	60.07 ± 0.33	60.02 ± 0.32

The statistical test of the model was performed using Fisher’s statistical method for analysis of variance (ANOVA). The result of ANOVA for *W*_C_, as shown in Table 6, implied a good agreement between the experimental and predicted data because the regression coefficient *R*^2^ reached 0.9585. The Model *F*-value was 17.97 and the value of “Prob > *F* = 0.0005” was less than 0.05, indicating significance of the model. Moreover, the total determination coefficient *R*^2^ value of 0.9052 demonstrated that only 9% of the total variation could not be explained by the model. Therefore, the mathematical model (Eq (4)) was able to describe the effects of Fenton’s reagent combined with DDBAC on sludge dewaterability and predict the *W*_C_ during sludge conditioning.

**Table 6:** Analysis of variance (ANOVA) for the quadratic model for *W*_C_.

Source	Sum of squares (SS)	Degrees of freedom (DF)	Mean square (MS)	*F*-value	*P* (Prob > *F*)
**Model**	78.62	9	8.74	17.97	0.0005
***X***_**1**_	7.41	1	7.41	15.24	0.0059
***X***_**2**_	4.77	1	4.77	9.82	0.0165
***X***_**3**_	4.09	1	4.09	8.41	0.023
***X***_**1**_^**2**^	16.66	1	16.66	34.26	0.0006
***X***_**2**_^**2**^	38.89	1	38.89	79.98	<0.0001
***X***_**3**_^**2**^	1.80	1	1.80	3.70	0.0957
***X***_**1**_***X***_**2**_	0.26	1	0.26	0.53	0.4883
***X***_**1**_***X***_**3**_	0.0016	1	0.0016	0.0033	0.9559
***X***_**2**_***X***_**3**_	0.0081	1	0.0081	0.017	0.9009
**Residual**	3.40	7	0.49		
**Lack of fit**	3.31	3	1.10	45.03	0.0015
**Pure error**	0.098	4	0.024		
**Cor. total**	82.02	16			

*R*^2^ = 0.9585; *R*^2^_adj_ = 0.9052; adequate precision = 13.123(>4)

From Eq (4), the minimal coded values of independent variables were determined using the Mathematical software and response surface analysis as follows: *X*_1_ = 0.23, *X*_2_ = − 0.12 and *X*_3_ = 0.55. Accordingly, H_2_O_2_, Fe^2+^, and DDBAC dosages were 44.6, 39.6, and 71.0 mg/g, at which the predicted value of the *W*_C_ was estimated to be 59.67%.

Considering these optimal conditions, three additional experiments were performed to validate the accuracy and practicability of the established polynomial equation. The replicated experiments yielded an average *W*_C_ of 59.54% ± 0.37%, which showed high agreement with the predicted value. Therefore, the quadratic model optimized the joint conditioning processes effectively.

### Results of mechanism

#### Sludge dewatering performance

**Fig 3:**
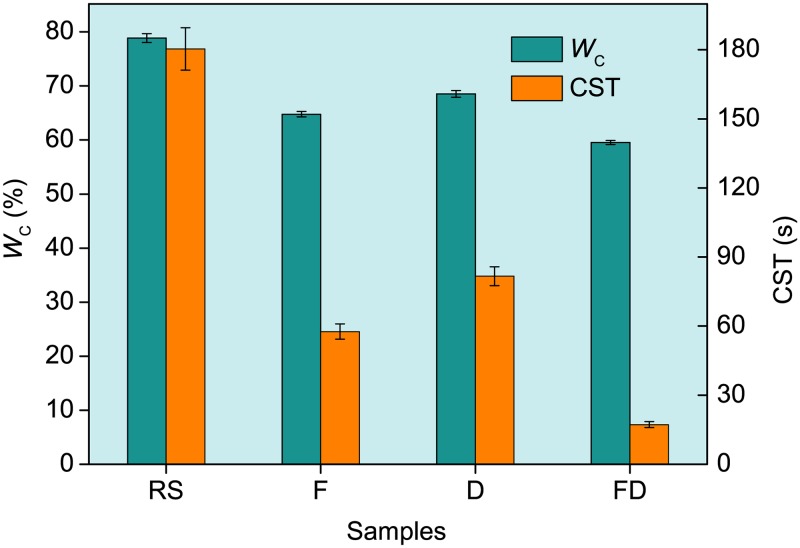
Effects of different conditioning situations on *W*_C_ and CST. RS, Raw sludge; F, Sludge conditioned using Fenton’s reagent; D, Sludge conditioned using DDBAC; FD, Sludge conditioned using Fenton’s reagent and DDBAC.

As shown in Fig 3, *W*_C_ and CST of raw sludge were 78.87% and 180.4 s, respectively. The *W*_C_ (59.54%) and CST (17.2 s) of the sludge conditioned using the composite conditioner were lower than those of the sludge conditioned using Fenton’s reagent alone or DDBAC alone, suggesting that the effect of joint conditioning was better than individual usage.

#### EPS changes in sludge

**Fig 4:**
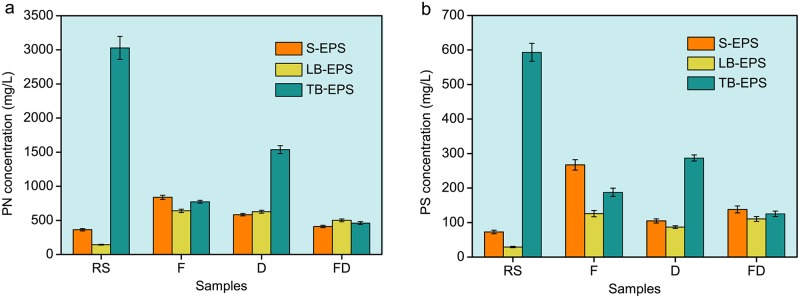
Effects of different conditioning situations on EPS. a Distributions and changes of PN in EPS layers. b Distributions and changes of PS in EPS layers. RS, Raw sludge; F, Sludge conditioned using Fenton’s reagent; D, Sludge conditioned using DDBAC; FD, Sludge conditioned using Fenton’s reagent and DDBAC.

As shown in Fig 4, the variations of PN and PS concentrations in S-EPS, LB-EPS, and TB-EPS were very obvious during conditioning. For the raw sludge, the PN and PS were mainly distributed in TB-EPS, accounting for 85.72% of total PN and 85.37% of total PS. The PN and PS in LB-EPS and S-EPS were less distributed. These findings agree with those in the previous literature [27], which demonstrated that more than 80% of EPS existed in the form of TB-EPS. For sludge conditioned using Fenton’s reagent, the PN concentration in TB-EPS decreased from 3028.17 to 773.18 mg/L, whereas the PN concentration in LB-EPS increased from 143.16 to 639.83 mg/L, and those in S-EPS increased from 363.31 to 838.86 mg/L (Fig 4a). Furthermore, the PS variation trend was similar to that of PN (Fig 4b). These results indicated that Fenton oxidation could effectively degrade EPS into dissolved organics that could be released into the filtrate [28–30].

After Fenton reaction, adding DDBAC further conditioned the sludge. The PN and PS concentrations in TB-EPS and S-EPS decreased, whereas the PN and PS concentrations in LB-EPS were almost unchanged. This trend may be because DDBAC weakened the surface tension of sludge and promoted the solubility of the non-dissolvable EPS. Therefore, a portion of insoluble EPS (TB-EPS and LB-EPS) was turned into soluble EPS (S-EPS) and was released into the liquid phase. However, the S-EPS hydrolysis was stronger than the solubilization of TB-EPS and LB-EPS under the action of DDBAC because most EPS had been peeled off, thereby decreasing S-EPS concentration.

#### Bound water content

Bound water is deeply embedded in sludge flocs due to intermolecular forces and cannot be removed mechanically, and this influences sludge dewaterability. Colin and Gazbar [31] reported that *W*_B_ could be directly used to measure the difficulty of mechanical dewatering. To be specific, a high *W*_B_ will cause mechanical dewatering difficulty, and vice versa.

**Fig 5:**
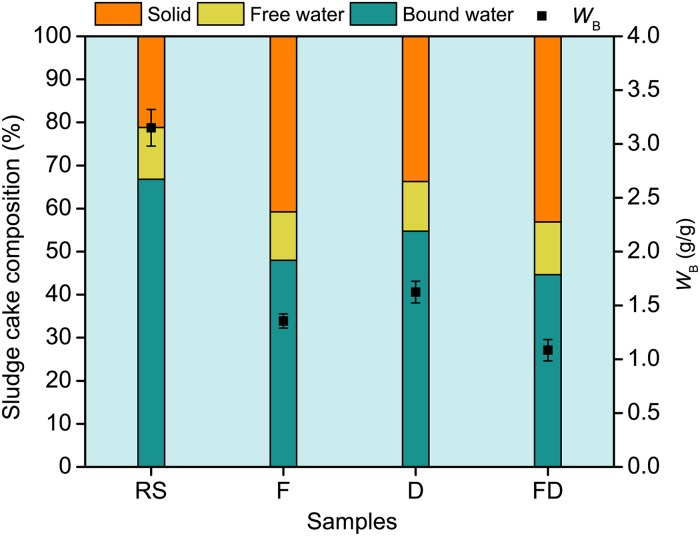
*W*_B_ and composition distribution in the sludge cake during conditioning. RS, Raw sludge; F, Sludge conditioned using Fenton’s reagent; D, Sludge conditioned using DDBAC; FD, Sludge conditioned using Fenton’s reagent and DDBAC.

Fig 5 shows *W*_B_ and sludge cake composition of the raw and conditioned sludge. The *W*_B_ of sludge conditioned using Fenton’s reagent decreased from 3.15 to 1.36 g/g and further decreased to 1.08 g/g after the sludge was jointly conditioned using Fenton’s reagent and DDBAC. Approximately 68% of the bound water was transformed into free water. The results were in good agreement with the sludge dewaterability, as depicted in Fig 3, which demonstrated that the high *W*_B_ in sludge causes poor sludge dewaterability. These findings are consistent with the conclusion of Kwon et al. [32] that stated a negative correlation between *W*_B_ and sludge dewatering performance.

The sludge cake composition showed that approximately 45% of the bound water in the conditioned sludge still remained, which could be because the water combined with sludge flocs through strong chemical bond and could not be released into the liquid phase by composite conditioner conditioning. The proportion of free water in the sludge cake had no significant change and remained within 11%–13% during conditioning, indicating that the free water removal rate depended on mechanical dehydrating units and had no correlation with sludge conditioning.

#### Organic acids concentration changes in the sludge supernatant

**Fig 6:**
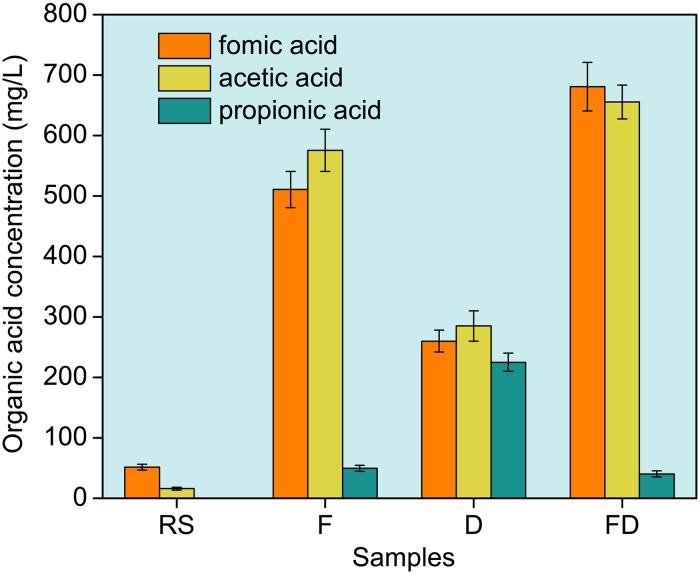
Variations of organic acid concentrations in the sludge supernatant. RS, Raw sludge; F, Sludge conditioned using Fenton’s reagent; D, Sludge conditioned using DDBAC; FD, Sludge conditioned using Fenton’s reagent and DDBAC.

Fig 6 shows that the three organic acid concentrations in the sludge supernatant, especially formic and acetic acid, markedly increased after conditioning. For the sludge conditioned using Fenton’s reagent, the formic and acetic acid concentrations increased to 535.72 and 575.42 mg/L, respectively. This finding further indicated that EPS was effectively degraded because of strong oxidation reactions and released into the liquid phase, which was conducive to the release of the bound water. After the Fenton reaction, the formic and acetic acid concentrations further increased with the addition of DDBAC. The results verified that the PN and PS in S-EPS were resolved into low-molecular-mass organics due to DDBAC hydrolysis, which was similar to the findings reported by Hong et al. [18, 19] and Huang et al. [33].

## Conclusions

Fenton’s reagent conditioning integrated with DDBAC significantly improved sludge dewatering performance when the sludge pH value was 4. The optimum dosages, determined by RSM, of the composite conditioner in this study were 44.6 mg/g H_2_O_2_, 39.6 mg/g Fe^2+^, and 71.0 mg/g DDBAC, at which 59.67% *W*_C_ was achieved. Three additional experiments at optimal conditions yielded an average *W*_C_ of 59.54% ± 0.37%, which showed high agreement with the predicted value.

The mechanism investigations showed that the composite conditioner could effectively decompose EPS into dissolved organics, resulting in the conversion of the bound water into free water. The *W*_B_ of the sludge conditioned using Fenton’s reagent decreased from 3.15 to 1.36 g/g and further decreased to 1.08 g/g after the sludge was jointly conditioned using Fenton’s reagent and DDBAC.
